# Tailoring the Properties and Oxidative Stability of *Idesia polycarpa* Crude Oil-Based HIPEs via Xanthan Gum and Ovalbumin: Implementation in Biscuit Processing

**DOI:** 10.3390/foods15101740

**Published:** 2026-05-14

**Authors:** Xiufang Huang, Yifan Shi, Yaobing Chen, Jianquan Kan, Kai Luo

**Affiliations:** 1 College of Biological and Food Engineering, Hubei Minzu University, Enshi 445000, China; 2015069@hbmzu.edu.cn (X.H.); 202430416@hbmzu.edu.cn (Y.S.); 1990024@hbmzu.edu.cn (Y.C.); 2 Hubei Key Laboratory of Biologic Resources Protection and Utilization, Hubei Minzu University, Enshi 445000, China; 3 College of Food Science, Southwest University, Chongqing 400715, China

**Keywords:** *Idesia polycarpa* crude oil, ovalbumin, high-internal-phase emulsion

## Abstract

This study aims to improve the utilization of *Idesia polycarpa* crude oil (IPCO) in the food industry by developing high-internal-phase emulsions (HIPEs) stabilized through ternary complexes (ovalbumin (OVA), xanthan gum (XG), and tannic acid (TA)). IPCO is highly prone to oxidation due to its polyunsaturated fatty acid (PUFA) content. Optimal formulations were obtained by varying the component concentrations and assessing the structure, stability, and fat-substitution potential. Under conditions of 0.6% *w*/*v* XG and 2.5% *w*/*v* OVA-TA, HIPEs exhibited a smaller particle size (3.31 μm), high centrifugal oil retention (99.29%), strong emulsifying activity (49.91 m^2^/g), and excellent stability (99.69%). Additionally, a formulation with 1.5% *w*/*v* OVA-TA and 0.8% *w*/*v* XG showed good wettability, particle size, and stability, possibly due to excessive self-aggregation of XG, which caused a decrease in emulsion stability and wettability. Structural analysis (FTIR, XRD, SEM, CLSM) revealed that the stability of the emulsions was mainly attributed to strong non-covalent interactions and a dense interfacial adsorption layer. In cookie applications, substituting 25% *w*/*w* butter or 50% *w*/*w* shortening with HIPEs resulted in comparable texture to the control group. GC–MS analysis of relative fatty acid composition showed that partial replacement with IPCO-based HIPEs shifted the final biscuits toward a lower relative proportion of palmitic acid (C16:0) and a higher relative proportion of linoleic acid (C18:2n6c). Overall, OVA–TA–XG-stabilized HIPEs effectively delayed the oxidation of IPCO and enabled partial replacement of conventional solid fats in biscuits, thereby shifting the relative fatty acid composition of the final products toward a higher proportion of unsaturated fatty acids.

## 1. Introduction

Current dietary recommendations emphasize reducing the intake of saturated fatty acids (SFAs) and industrially produced trans fatty acids (TFAs), as excessive consumption of these fats is associated with an increased risk of diet-related noncommunicable diseases, including cardiovascular disease and type 2 diabetes. Accordingly, replacing fats rich in SFAs or TFAs with unsaturated vegetable oils has been recognized as an important dietary strategy for improving lipid profiles and promoting metabolic health [1].

In bakery products, however, conventional solid fats such as butter and shortening provide essential technological functions, including plasticity, lubrication, aeration, shortening effects, and texture formation [2,3,4,5]. Direct replacement of these solid fats with liquid vegetable oils often compromises dough handling properties and final product quality. Traditionally, hydrogenation has been used to increase the solidity and plasticity of vegetable oils, but this process may generate trans fatty acids and raise health concerns [6]. Therefore, developing structured lipid systems that can mimic the functionality of solid fats while incorporating unsaturated vegetable oils has become an important approach for improving the nutritional quality of bakery products.

Consequently, the development of novel fat structuring technologies that combine solid state functionality with nutritional benefits has become a major research focus. The critical challenge lies in solidifying liquid oils without producing trans fatty acids while simultaneously reducing the saturated fatty acid content in the final products. Recently, oleogelation technology has emerged as a promising strategy. By precisely modulating the crystalline network of oils, it preserves the processability and plasticity of solid fats, significantly enhances the retention of polyunsaturated fatty acids, and improves oxidative stability. This approach offers a breakthrough solution for building solid fat systems with both functional and nutritional advantages [7]. However, the applicability of oleogels is often constrained by the need for high concentrations of structuring agents, particularly for oils with specific unsaturated fatty acid profiles. In this context, high-internal-phase emulsions (HIPEs) offer an alternative approach. With an internal phase volume fraction exceeding 74%, HIPEs exhibit a semi solid texture and excellent encapsulation ability [8,9]. Compared with oleogels, HIPEs allow for greater flexibility in modulating texture and caloric density through water incorporation. They can be stabilized by protein polysaccharide complexes to enhance mechanical strength and oxidative stability [10,11]. For the specific oil used in this study, which is characterized by a high proportion of unsaturated fatty acids, HIPEs provide a more adaptable platform than oleogels. They do not rely on high concentrations of structuring agents and can be tailored to meet the rheological requirements of the target bakery product. Therefore, this study focuses on HIPEs as the structuring method of choice, rather than pursuing oleogelation, to achieve the desired lipid profile and functional properties.

Native to East Asia, the woody plant *Idesia polycarpa Maxim*. produces seeds with a high oil content and has been proposed as a promising bio-based feedstock. Its fruits have been reported to contain oil content ranging from 26.26% to 43.6% (*w*/*w*) [12]. In terms of oil yield per unit area, some estimates suggest it can reach 2.25–3.75 tons per hectare under specific cultivation conditions, a range that has been described as potentially comparable to that of oil palm and substantially higher than conventional herbaceous oil crops [13,14]. However, these figures are context-dependent and may vary with region, agricultural practices, and genotype. The oil derived from *Idesia polycarpa* is nutritionally distinctive, being rich in polyunsaturated fatty acids (PUFAs, 64.01–71.72% *w*/*w*), with linoleic acid (63.07–70.69% *w*/*w*) as the predominant component. These levels are notably higher than those found in soybean oil (55.33% *w*/*w*) and olive oil (12.48% *w*/*w*) [12]. Toxicological and long-term dietary safety evaluations have confirmed its suitability for human consumption [15,16]. Nevertheless, owing to its high PUFA content, *Idesia polycarpa* crude oil (IPCO) is highly prone to oxidation, resulting in poor stability and rapid quality deterioration. Regarding its agronomic characteristics, the species is reported to exhibit fast growth, with some studies indicating an annual height increment of 3–5 m, and wide ecological adaptability, growing at altitudes ranging from 500 to 3000 m depending on local conditions [17]. These attributes, however, are influenced by environmental factors and cultivation practices rather than being fixed traits. Thus, while the species combines potentially high oil yield, rapid growth, broad adaptability, and nutritionally valuable oil production, the oxidative instability of its crude oil remains a major bottleneck that must be addressed to realize its potential in developing a novel functional lipid industry.

Research on the construction of HIPEs based on IPCO remains limited. Existing studies have successfully developed protein–polyphenol-stabilized HIPE systems (e.g., ovalbumin (OVA)–tannic acid (TA) complexes), demonstrating their effectiveness in structuring edible oils and enhancing oxidative stability through interfacial engineering [18]. However, these studies typically employ fixed component ratios, which elucidate the synergistic effects of combined stabilizers but do not clarify the individual contribution of each component to system performance. Furthermore, the application of such strategies to highly unsaturated oils such as IPCO remains unexplored, and the role of polysaccharide addition (e.g., xanthan gum) in modulating the microstructure and rheological properties of OVA–TA-stabilized HIPEs has not been systematically investigated. To address these gaps, this study developed an IPCO-based HIPE system stabilized by OVA–TA–XG complexes using a one-step fabrication method. It should be noted that the TA:OVA mass ratio was fixed at 1:10 based on preliminary optimization; therefore, the individual contributions of OVA and TA cannot be dissociated within the current experimental design. The specific objectives of this study were: (i) to optimize the formulation of IPCO-based HIPEs by varying the concentrations of OVA–TA complexes and XG; (ii) to elucidate the structure–function relationships among complex characteristics, droplet structure, rheological properties, oxidative stability, and emulsion performance; and (iii) to evaluate the feasibility of the optimized HIPEs as partial substitutes for butter and shortening in biscuit processing.

## 2. Materials and Methods

### 2.1. Materials and Chemicals

*Idesia polycarpa Maxim* was purchased from Jianshi County, Enshi City, Hubei Province (Enshi, China). Ovalbumin (purity ≥ 98% *w*/*v*) was purchased from Shanghai Macklin Biochemical Technology Co., Ltd. (Shanghai, China). The following chemicals were obtained from Shanghai Aladdin Biochemical Technology Co., Ltd. (Shanghai, China): xanthan gum, tannic acid (purity ≥ 98% *w*/*v*), boron trifluoride–methanol complex, and n-hexane. Isooctane, isopropanol, ether, and petroleum ether were all purchased from Sinopharm Co., Ltd. (Beijing, China). 37 Fatty Acid Mixed Standards were purchased from Sigma-Aldrich Co. LLC. (St. Louis, MO, USA). Commercially available salt, white sugar, low-gluten wheat flour, fresh eggs, and butter were used.

### 2.2. Preparation of Idesia polycarpa Crude Oil (IPCO)

Select fresh *Idesia polycarpa* seeds, remove defective ones, and dry them in a 55 °C hot air pump drying oven (Boxun Medical Biological Instrument Corp., Shanghai, China) until a constant weight is reached. The seeds were then separated from the dried fruits, and oil was extracted from the seeds using an oil press. The obtained crude oil was centrifuged at 4200× *g* (Heal Force Bio-Meditech Holdings Ltd., Shanghai, China) for 20 min to obtain pure seed oil. The oil yield from seeds was approximately 24% (*w*/*w*). The extracted oil was stored in amber glass bottles flushed with nitrogen to minimize light exposure and oxidation, and kept at 4 °C until use. All experiments were conducted within two weeks after oil extraction to ensure minimal oxidative deterioration.

### 2.3. Preparation of OVA-TA-XG Complex Solution

This study followed the method of Yan et al. [18] to construct an ovalbumin–tannic acid–xanthan gum composite system. It should be noted that the TA:OVA mass ratio was fixed at 1:10 based on preliminary optimization, and TA was not independently varied in the present study. In addition, OVA-only, OVA–XG, and OVA–TA control systems were not included. Therefore, the individual contributions of OVA, TA, and XG cannot be fully dissociated, and the results should be interpreted as the combined effects of the OVA–TA complex and XG under the selected formulation conditions. Under the conditions employed in this study (pH 7.0, storage at 4 °C), the interaction between OVA and TA is primarily attributed to non-covalent forces (such as hydrogen bonding and hydrophobic interactions), rather than covalent grafting. The specific preparation process is as follows: First, gradient concentrations of OVA aqueous solutions (0.5–2.5% *w*/*v*, in increments of 0.5% *w*/*v*) were prepared; these represent pre-mixing concentrations. After homogenization at 100 rpm for 2 h, TA was added to the OVA solution at a TA:OVA mass ratio of 1:10, and the stirring speed was increased to 240 rpm for a continuous 2 h reaction. The system pH was titrated to 7.0 using 0.1 M HCl or NaOH, and the solution was then stored quiescently at 4 °C for 12 h to facilitate complexation. The mixture was dialyzed against deionized water at 4 °C using a 3500 Da molecular weight cutoff dialysis bag for 48 h (changing the water every 8 h) to remove unbound TA. XG solutions of different concentrations (0.2–1.0% *w*/*v*, in increments of 0.2% *w*/*v*) were prepared separately; these represent pre-mixing concentrations. After pre-hydration at 240 rpm for 2 h, they were mixed with the OVA–TA system at a 1:1 volume ratio. When the final XG concentration in the mixed system was fixed at 0.6% *w*/*v*, the effect of OVA concentration (pre-mixing concentration 0.5–2.5% *w*/*v*, corresponding to a final concentration of 0.25–1.25% *w*/*v* after 1:1 dilution) on complexation was investigated; when the final OVA concentration was fixed at 1.5% *w*/*v* (corresponding to a pre-mixing concentration of 3.0% *w*/*v*), the compatibility of XG concentrations (pre-mixing concentration 0.2–1.0% *w*/*v*, corresponding to a final concentration of 0.1–0.5% *w*/*v* after dilution) was examined. The final composite system was stirred continuously at 25 °C for 12 h to complete formation. Preparation of OVA–TA–XG composite particles: during the composite particle preparation stage, the above composite solution was pre-frozen in an ultra-low temperature freezer at −80 °C for 48 h, then transferred to a freeze dryer and lyophilized for 48 h to form composite particles for analysis.

### 2.4. OVA-TA-XG Composite Particle Evaluation System

#### 2.4.1. OVA–TA Interaction Analysis

The SDF (Structure Data File) and crystal structures of tannic acid and ovalbumin were obtained from PubChem https://pubchem.ncbi.nlm.nih.gov/ (accessed on 10 November 2025) and the PDB https://www.rcsb.org/ (accessed on 10 November 2025). Water molecules and original ligands were removed from the target protein using PyMOL 3.11 to expose the active site. Chem3D 23.1.1 was used to optimize the conformational energy of the small molecules. AutoDockTools 1.5.7 was used to process the receptor (hydrogenation) and the ligands (rotatable bonds). PDBQT files were generated using AutoDock Vina 1.2.3. Molecular docking with semi-flexible ligands was performed using AutoDock Vina (v1.2.3). The grid size was defined by the binding coordinates of the crystallographic ligand, and the exhaustiveness was set to 10 to optimize the conformational search. Finally, the PyMOL online platform, together with PyMOL’s 3D visualization capabilities and analysis of key binding patterns such as hydrogen bonds and hydrophobic interactions, was used to elucidate ligand–receptor interaction mechanisms [19].

#### 2.4.2. X-Ray Diffraction Analysis

Lyophilized OVA-TA-XG (XG-0.6% *w*/*v*, OVA-0.5–2.5% *w*/*v*) (OVA-1.5% *w*/*v*, XG-0.2–1.0% *w*/*v*) at different concentrations were analyzed using an X-ray diffractometer Bruker D2 Phaser X-ray diffractometer, Bruker AXS GmbH, Karlsruhe, Germany at a scan rate of 5°/min over a range of 5–60°.

#### 2.4.3. Scanning Electron Microscopy (SEM)

Composite particles of OVA-TA-XG (XG: 0.6% *w*/*v*, OVA: 0.5–2.5% *w*/*v*) (OVA: 1.5% *w*/*v*, XG: 0.2–1.0% *w*/*v*) were affixed to circular sample disks using double-sided tape and then gold-sprayed. Micrographs were obtained using a TESCAN MIRA LMS scanning electron microscope (TESCAN ORSAY HOLDING, a.s., Brno, Czech Republic).

#### 2.4.4. Determination of Wettability

The hydrophilicity or hydrophobicity of the samples can be determined by contact angle experiments. Specifically, a 10 μL water droplet is placed on OVA–TA–XG composite films of different concentrations, the droplet shape was first visualized with a contact angle instrument. The three-phase contact angle (θ) was then calculated by applying the Young–Laplace equation to the obtained images.

### 2.5. Preparation of OVA-TA-XG-Based HIPES

This study was modified based on the research plan of Qiu et al. [20]. The specific steps are as follows: The OVA–TA–XG composite particle solutions of different concentrations prepared above were mixed with IPCO at an oil phase volume fraction of 75% (φ = 0.75, based on the total emulsion volume). The emulsification was performed using a high-speed homogenizer (IKA T18 digital Ultra-Turrax, IKA-Werke GmbH & Co. KG, Staufen, Germany) equipped with a dispersing element (S18N-19G rotor–stator head, 19 mm diameter; IKA-Werke GmbH & Co. KG, Staufen, Germany). A batch size of 50 mL total emulsion volume was prepared in a 100 mL glass beaker. The oil phase was added gradually to the aqueous phase containing the composite particles over a period of approximately 60 s while homogenizing at low speed (4200 rpm) to facilitate preliminary mixing. Subsequently, the mixture was sheared at 24,500 rpm for 3 min to form a primary emulsion. Temperature was controlled by placing the sample vessel in an ice–water bath during homogenization to prevent overheating, and the emulsion was used immediately after preparation.

### 2.6. Characterization Methods for OVA–TA–XG-Stabilized HIPEs

#### 2.6.1. Optical Microscopy Particle Size Analysis

Following a 10-fold dilution, 10 μL of the emulsion was evenly spread on a glass slide, covered with a coverslip, and examined under a microscope. Morphological observations were made using a BM2100POL (T/R) polarizing microscope (Ningbo Yongxin Optics Co., Ltd., Ningbo, China) equipped with a digital camera. For droplet size analysis, at least five random fields of view were selected from each sample, and a minimum of three images were captured per field. The droplet diameter distribution was analyzed using ImageJ software 1.54j (National Institutes of Health, Bethesda, MD, USA). A total of at least 200 droplets were measured per sample to ensure statistical representativeness. Droplet boundaries were identified using the default automatic thresholding method in ImageJ, with manual correction applied when necessary to ensure accurate segmentation.

#### 2.6.2. Confocal Laser Scanning Microscopy (CLSM) Observation

After the samples were allowed to rest in centrifuge tubes, the entire emulsion was gently mixed by inverting the tube several times to ensure homogeneity. Then, 1 mL of the emulsion was transferred from each sample into a 2 mL centrifuge tube. To each tube, 20 μL of a mixed staining solution (Nile Blue, 20 μg/mL; Nile Red, 20 μg/mL; Yuanye Bio-Technology Co., Ltd., Shanghai, China) was added, vortexed to mix, and incubated at 37 °C for 30 min. After staining, 20 μL of the sample solution was placed on a glass slide, covered with a coverslip, and imaged using a laser scanning confocal microscope (100×, 200×; ZEISS LSM 900, Carl Zeiss Microscopy GmbH, Jena, Germany). Nile Red was excited with a 561 nm laser and detected over the 450–645 nm wavelength range (displayed in red); Nile Blue was excited with a 640 nm laser and detected over the 645–700 nm wavelength range (displayed in blue).

#### 2.6.3. Centrifugal Oil Retention

The methodology was adapted from Xu et al. [21] with modifications. Briefly, HIPE samples with different OVA–TA–XG concentrations were centrifuged at 6100× *g* for 15 min. After centrifugation, the tubes were inverted, and the released oil was carefully removed using filter paper. The following Equation (1) was used to calculate centrifugal oil retention.

(1) $$Centrifugal\;oil\;retention(\%)=\frac{\left(m_{2}-m\right)}{\left(m_{1}-m\right)}\times 100$$

In this equation, *m*_1_ and *m*_2_ denote the masses of the centrifuge tube containing the sample before and after centrifugation, respectively, and *m* denotes the mass of the empty centrifuge tube.

#### 2.6.4. Auxiliary Emulsifying Properties After Dilution

EAI and ESI were determined as auxiliary indicators of the emulsifying capacity of the OVA–TA–XG systems after dilution, rather than as definitive measures of HIPE stability. Based on the methods of Huang et al. [22] and Cameron et al. [23], the sample solution was mixed with IPCO at a 3:1 (*v*/*v*) ratio. Following collection of a 100 μL sample from the bottom phase of the emulsion, it was uniformly dispersed in 3.0 mL of SDS solution (0.1% *w*/*w*, pH 7.0). The absorbance A_0_ of the fresh emulsion and A_10_ of the emulsion after 10 min of standing were measured at 500 nm. EAI and ESI were calculated according to the following equations:

(2) $$EAI\left(m^{2}/g\right)=\frac{2\times 2.303\times A_{0}\times DF}{C\times \phi \times L\times 10^{4}}$$

(3) $$ESI\left(\%\right)=\frac{A_{10}}{A_{0}}\times 100$$

In this equation, *DF* represents the emulsion dilution factor and *C* is the protein concentration (g/mL) in the aqueous solution. The oil phase volume fraction is denoted by *φ*, while *L* is the fixed optical path length of 1 cm; *A*_0_ and *A_1_*_0_ denote the absorbance values of the emulsion at time 0 and 10 min. These measurements were conducted at a wavelength of 500 nm.

#### 2.6.5. Measurement of Rheological Properties

The rheological properties of the emulsions were measured using a rotational rheometer equipped with a 40 mm diameter parallel plate geometry, with a gap set to 1.0 mm. All measurements were performed at 25 °C. The sample was carefully loaded onto the lower plate, and the upper plate was lowered to the measuring gap position. Excess sample extending beyond the plate edge was trimmed carefully, and a solvent trap was used to minimize evaporation during measurements. The sample was allowed to rest for 5 min after loading to ensure thermal equilibration and structure recovery before initiating the tests. First, an oscillatory stress (τ) sweep (τ = 1–100 Pa) was performed at a fixed frequency (f) of 1 Hz to determine the linear viscoelastic region (LVR). Based on the LVR results, a constant stress of 3 Pa (within the LVR) was selected for the subsequent frequency sweep. An oscillatory frequency sweep was then conducted over a frequency range of 0.1–10 Hz at this constant stress of 3 Pa. The corresponding storage modulus (G′) and loss modulus (G″) were recorded. Finally, the effect of shear rate (0.01–100 s^−1^) on the apparent viscosity of the emulsion gel was evaluated under steady shear conditions.

#### 2.6.6. Stability Tests Under Storage, Heating, and Freeze–Thaw Conditions

The following protocols were employed to evaluate the stability of the emulsions, recording both visual observations and quantitative indicators. Thermal stability assessment: Freshly prepared emulsion samples (10 mL) were sealed in glass bottles, placed in a water bath at 80 °C for 30 min, and then cooled to room temperature (25 °C) for 1 h. Stability was evaluated by visually observing phase separation. Storage stability assessment: Emulsion samples (10 mL) were sealed in glass bottles and stored at 4 °C for 28 days. The samples were visually inspected for creaming or phase separation weekly (on days 0, 7, 14, 21, and 28). Freeze–thaw stability assessment: Emulsion samples (10 mL) were frozen at −20 °C for 24 h and then thawed at room temperature (25 °C) for 2 h. This freeze–thaw cycle was repeated three times. After each cycle (1st, 2nd, and 3rd), the samples were photographed to document visual changes.

#### 2.6.7. Measurement of Oxidative Stability

The oxidative stability of the prepared HIPEs were assessed using an accelerated oxidation test. Both the HIPEs and the crude IPCO sample were subjected to incubation at 60 °C in the dark. Oxidation indices, including lipid hydroperoxides quantified by the ferric thiocyanate method, were measured at predetermined intervals (days 0, 2, 4, 6, 8, 10, and 12) [24]. The phases were first separated via centrifugation at 3600× *g* for 2 min using a swinging-bucket rotor. Subsequently, 200 μL of the upper organic phase was mixed with 2.8 mL of methanol/n-butanol (2:1, *v*/*v*). To this mixture, 50 μL each of 0.3 g/mL ammonium thiocyanate and 28.6 mg/mL ferrous chloride solutions were added. After allowing the reaction to proceed for 10 min, the absorbance was recorded at 510 nm. Hydroperoxide content was calculated using a cumene hydroperoxide standard curve, and each determination was performed in triplicate.

In accordance with a modified colorimetric assay, an accurately weighed 0.2 g sample was subjected to reaction with a mixed reagent consisting of 15% *w*/*v* trichloroacetic acid, 0.375% *w*/*v* thiobarbituric acid, and 2% *w*/*v* HCl [25]. The mixed system was subjected to a 20-min treatment in a 90 °C water bath to enable complete formation of the characteristic pigments. To remove the precipitate, the mixture was first cooled to ambient temperature (25 °C) and then centrifuged at 3500× *g* for 15 min. The absorbance of the supernatant was measured at 532 nm, and the malondialdehyde equivalent concentration was calculated using a 1,1,3,3-tetraethoxypropane standard curve. The experiment was repeated three times independently.

### 2.7. Application of OVA-TA-XG HIPES in Biscuits

#### 2.7.1. Preparation of Biscuit Dough

The dough was prepared according to the method of Gao et al. [26] with slight modifications. Fat replacement was performed on a mass basis, where HIPE directly replaced butter/shortening at levels of 0% (control, 100% butter/shortening), 25% (25% HIPE + 75% butter/shortening), 50% (50% HIPE + 50% butter/shortening), 75% (75% HIPE + 25% butter/shortening), and 100% (full HIPE replacement) by weight of the original fat content. It is important to note that because HIPEs contain a significant amount of water (approximately 25% *w*/*w* in the aqueous phase), mass-based replacement alters both the fat and water content of the dough compared to the control. To maintain constant dough consistency and isolate the effect of fat replacement, the total water addition was adjusted across formulations to achieve equivalent final dough hydration. This approach ensures that observed differences in texture and baking loss can be attributed to the fat structure and composition rather than to variations in dough moisture content. First, sugar (30 g) and the fat component (butter, shortening, or HIPEs with butter/shortening mixture at the specified replacement levels, total 40 g) were kneaded for 5 min. Then, whole egg liquid (45 g) was added in two portions and kneaded for another 3 min. Finally, flour (100 g) was added and kneaded for 3 min. The kneaded dough was left to rest at room temperature for 15 min to eliminate internal stress. The dough was then sheeted to a uniform thickness of 5 cm, cut into circular pieces of 10 cm diameter (approximately 40 g per piece), and placed on a baking tray lined with parchment paper. Baking was performed in a preheated domestic electric oven (Midea Group Co., Ltd., Foshan, China) using a two-stage temperature program: first at 120 °C (top and bottom heat) for 10 min, followed by 240 °C (top and bottom heat) for 20 min. After baking, the cookies were removed from the oven and cooled on a wire rack to room temperature (25 °C) for 1 h before further analysis. All baking experiments were performed in triplicate.

#### 2.7.2. Baking Loss Rate

The baked dough was weighed and controlled to be 40 g. The baking loss rate was calculated according to the following formula (4):

(4) $$Baking\;loss\;rate\;\left(\%\right)=\left(1-\frac{m}{40}\right)\times 100$$

#### 2.7.3. Biscuit Texture Analysis

A texture analyzer was employed to determine the biscuit texture, adapting the protocol of Song et al. [27] with minor adjustments. Briefly, a P50 cylindrical probe was used. The following settings were used: a pre-test, test, and post-test speed of 2.0, 1.0, and 2.0 mm/s, respectively; a target force of 250 g; and a compression interval of 5 s.

#### 2.7.4. Color Measurement of Biscuits

The color of the biscuits was analyzed using an automatic colorimeter (Konica Minolta CR-400, Konica Minolta, Inc., Tokyo, Japan) following the method of Felisiak et al. [28]. Prior to measurement, the colorimeter was calibrated using a standard white plate (20933026). Color measurements were performed using the CIE L*a*b* color space, with a D65 illuminant, a 10° observer angle, and an 8 mm aperture. The L* (lightness), a* (red-green value), and b* (yellow-blue value) values were recorded. Each sample was measured three times at different positions, and the average value was calculated. Biscuits prepared with commercial shortening or butter served as control samples. The color difference (ΔE) was calculated using the following formula:

(5) $$\Delta E=\sqrt{{\left(L^{*}p-L^{*}c\right)}^{2}+{\left(a^{*}p-a^{*}c\right)}^{2}+{\left(b^{*}p-b^{*}c\right)}^{2}}$$

where L*p, a*p, b*p are the color coordinates of OVA-TA-XG HIPE cookies; L*c, a*c, b*c, those of butter/shortening-based cookies.

#### 2.7.5. GC-MS Analysis of Fat Profile in Biscuits

The fatty acid composition of the biscuits was determined according to the method of Onacik-Gür et al. [29]. A 2 g sample was weighed, and 10 mL of ethanol, 5 mL of water, and 5 mL of HCl were added, followed by hydrolysis in an 80 °C water bath for 40 min. The mixture was extracted twice with 25 mL of petroleum ether/ethyl ether (1:1, *v*/*v*). The organic phases were combined and evaporated to dryness under nitrogen. Approximately 50 mg of the obtained fat was subjected to methylation by sequentially adding 2 mL of 0.5 M sodium methoxide solution (heated at 45 °C for 5 min) and 2 mL of 14% boron trifluoride in methanol solution (heated at 45 °C for 3 min). After cooling, 2 mL of n-hexane and 5 mL of saturated sodium chloride solution were added, and the mixture was vortexed for 30 s. The upper n-hexane phase was collected, dried over anhydrous sodium sulfate, and filtered through a 0.22 μm syringe filter. Analysis of fatty acid methyl esters (FAMEs) was performed using a Trace 1310 ISQ GC-MS system (Thermo Fisher, Waltham, MA, USA) equipped with a TG-FAME column (50 m × 0.25 mm × 0.20 μm). The injection volume was 1 μL with a split ratio of 100:1. The injector temperature was 260 °C, and the helium carrier gas flow rate was 0.63 mL/min. The oven temperature program was as follows: initial temperature of 80 °C held for 1 min, increased to 160 °C at 5 °C/min, then increased to 230 °C at 8 °C/min and held for 5 min. Mass spectrometry was performed using electron ionization at 70 eV, with an ion source temperature of 280 °C, a transfer line temperature of 240 °C, and a solvent delay of 4 min. Identification was achieved by comparing retention times with a 37-component FAME mix standard. Since no internal standard was used, the fatty acid results were calculated using the peak area normalization method and should be interpreted as relative or semi-quantitative composition rather than absolute quantitative concentrations.

### 2.8. Statistical Analysis

All data were analyzed and visualized using OriginPro 2024 (OriginLab Corporation, Northampton, MA, USA). Continuous variables are expressed as mean ± SD. Normality was assessed using the Shapiro–Wilk test. For normally distributed data, one-way analysis of variance with Tukey’s post hoc test was used to assess between-group differences; nonnormally distributed data were analyzed using the Kruskal–Wallis test with Dunn’s post hoc correction. Statistical significance was assumed at the 95% level (*p* < 0.05).

## 3. Results

### 3.1. Evaluation of OVA-XG-TA Composite Particles

#### 3.1.1. OVA–TA Interaction and FTIR Analysis

**Figure 1:**
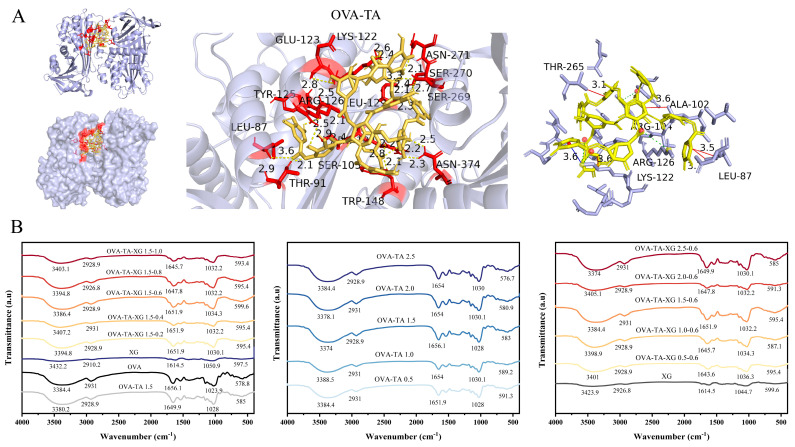
OVA–TA molecular docking and FTIR analysis of OVA–TA–XG complexes. (**A**) Molecular docking between OVA and TA; (**B**) FTIR spectra of OVA–TA–XG complexes at different concentrations. Data represent means ± SD (*n* = 3).

Molecular docking was used only as a supplementary tool to explore the possible interaction between OVA and TA (Figure 1A). The docking result suggested that TA could interact with OVA through hydrogen bonding, hydrophobic interactions, cation–π interactions, and salt bridges, with a predicted binding energy of −10.20 kcal/mol. These results indicate that TA may bind to OVA through multiple non-covalent interactions. In contrast, molecular docking was not applied to XG in the revised manuscript because XG is a high-molecular-weight and flexible polysaccharide that is not suitable for conventional docking analysis. Therefore, the role of XG in the OVA–TA–XG system was interpreted based on experimental evidence, including FTIR, XRD, SEM, wettability, droplet size, rheology, and stability results, rather than docking-derived binding energies or specific residue interactions.

In the infrared region of 3200–3500 cm^−1^, absorption peaks are typically associated with O–H/N–H stretching vibrations [30]. The changes in the broad O–H/N–H stretching region may suggest alterations in the hydrogen-bonding environment among OVA, TA, and XG. However, FTIR peak shifts alone cannot quantify hydrogen bonding strength, and these spectral changes may also be influenced by hydration state, concentration-dependent effects, molecular packing, or aggregation. Therefore, the FTIR results should be interpreted as indirect evidence of possible non-covalent interactions and should be considered together with XRD, SEM, wettability, droplet size, and rheological results. In this study, when the concentration of the OVA–TA complex was fixed at 1.5% *w*/*v* (with TA:OVA fixed at 1:10 based on preliminary optimization), increasing the XG concentration from 0.2% *w*/*v* to 1.0% *w*/*v* induced changes in both peak position and absorbance. At 0.4% *w*/*v* XG, the absorption peak was located at 3407 cm^−1^ with increased absorbance, likely due to the abundant hydroxyl groups in XG promoting hydrogen bond formation with OVA–TA. When the XG concentration was increased to 0.6% *w*/*v*, the absorption peak shifted to 3386.4 cm^−1^ with decreased absorbance, indicating enhanced hydrogen bonding interactions between XG and OVA–TA, which led to a reduction in the number of free hydrogen-bonding groups. However, upon further increasing the XG concentration beyond 0.6% *w*/*v*, the peak position shifted back to higher wavenumbers (3394.8 cm^−1^ at 0.8% *w*/*v* and 3403.1 cm^−1^ at 1.0% *w*/*v*) with increased absorbance, suggesting that excess XG may form a dense entangled network, thereby weakening hydrogen bonding interactions with OVA–TA and increasing the number of free hydroxyl groups. A similar trend was reported by Wang et al. for soybean protein isolate–xanthan gum-stabilized emulsion systems [31].

Conversely, when the XG concentration was fixed at 0.6% *w*/*v* and the OVA–TA complex concentration was varied (with TA:OVA fixed at 1:10), the peak position and absorbance exhibited non-linear changes, shifting to the lowest wavenumber of 3374.0 cm^−1^ at 2.5% *w*/*v* OVA–TA. This pattern suggests that hydrogen bonding interactions between the two components might be strongest at this concentration. Furthermore, the shifts and broadening of the stretching vibration bands confirmed the reorganization of the hydrogen bonding environment and the formation of new hydrogen bond networks among proteins, tannins, and polysaccharides [32,33]. Corresponding shifts in the amide I band (1643.6–1651.9 cm^−1^), most pronounced at XG = 1.0% *w*/*v* (1645.7 cm^−1^) and OVA:TA = 2.5% *w*/*v* (1643.6 cm^−1^), provided further evidence of protein secondary structure perturbation [34]. Simultaneously, the shift in the polysaccharide fingerprint region from 1044 cm^−1^ (XG control) to 1030–1036 cm^−1^ confirmed the involvement of C–O and C–O–C groups in protein–polyphenol interactions [35].

It should be noted that within this experimental design, TA was varied concomitantly with OVA at a fixed ratio of 1:10; therefore, while the overall effect of the OVA–TA complex can be assessed, the individual contributions of OVA and TA to the observed spectral changes cannot be distinguished. To fully elucidate single-component effects, future studies employing experimental designs with independently varied TA concentrations, as well as control systems without TA and/or XG, are necessary. No clear new absorption peaks associated with covalent bond formation were observed in the 1700–1730 cm^−1^ region under the present experimental conditions. However, the absence of new peaks in this region does not definitively exclude possible covalent interactions. More direct evidence, such as SDS-PAGE, free amino group analysis, or mass spectrometry, would be required to confirm whether covalent bonding occurred [36]. Collectively, these spectral changes suggest that the complex is primarily stabilized by non-covalent interactions—hydrogen bonding, electrostatic forces, and hydrophobic effects—consistent with previous findings on protein–polyphenol and protein–polysaccharide complexes [36]. In summary, by finely modulating the XG concentration and the OVA:TA ratio, the structural organization and interaction strength within the protein–polyphenol–polysaccharide complex can be tuned, providing a theoretical basis for the rational design and optimization of its functional properties (Figure 1B).

#### 3.1.2. X-Ray Diffraction Analysis and SEM Imaging

**Figure 2:**
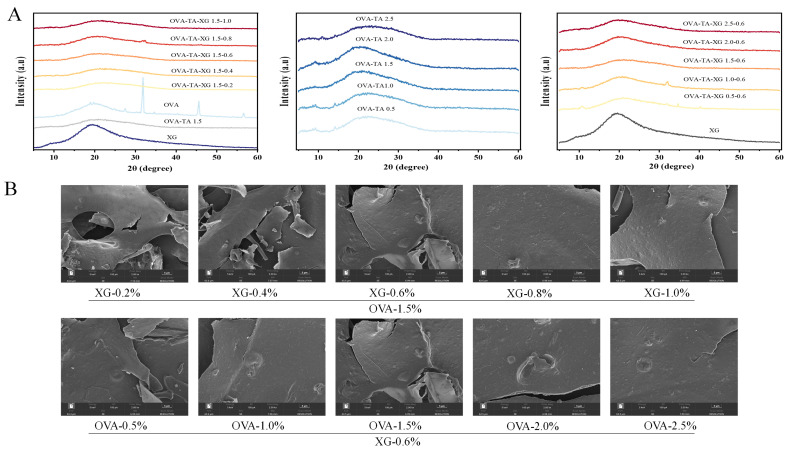
XRD and SEM analyses of composite particles at different concentrations. (**A**) XRD patterns of composite particles; (**B**) SEM images of composite particles. Data represent means ± SD (*n* = 3).

With XG fixed at 0.6% *w*/*v*, increasing the OVA–TA concentration from 0.5% *w*/*v* to 2.5% *w*/*v* led to noticeable changes in the XRD patterns (Figure 2A). The broad diffraction feature near 20° became more evident, while the sharp diffraction peaks associated with OVA gradually weakened or disappeared. These changes may indicate alterations in molecular packing, ordered domains, or aggregation states within the OVA–TA–XG complexes, rather than definitive evidence of a complete crystalline–amorphous transition. This interpretation is consistent with the FTIR amide I band observations, which showed shifts, broadening, and intensity changes, suggesting possible changes in the hydrogen-bonding environment and protein secondary structure. Mechanistically, the interaction between TA and OVA, mainly driven by hydrogen bonding and hydrophobic interactions, may contribute to partial structural rearrangement of OVA and the formation of dispersed TA–protein complexes, thereby reducing some pre-existing ordered domains, as reported in previous studies [37,38]. Although competitive interactions between TA and protein chains have been proposed [39,40], this mechanism may be less dominant in the present system because the OVA:TA ratio remained constant. Instead, the observed changes appear to be more closely associated with variations in OVA–TA loading and XG concentration. Moderate XG levels may help stabilize the OVA–TA complexes by affecting solution viscosity, steric effects, and molecular association, whereas excessively high XG concentrations may induce local phase separation or OVA enrichment, leading to partial reappearance of OVA-related diffraction features. This explanation is consistent with Liu et al., who reported that appropriate XG addition influenced the secondary structure of SPI, whereas excessive polysaccharide promoted phase separation and structural disorder [41]. Overall, the XRD and FTIR results suggest that OVA–TA loading and XG concentration may jointly influence the molecular ordering, packing, and aggregation state of the complexes, which may further affect interfacial behavior, droplet size, and emulsion stability. However, these results should be interpreted as indirect evidence of structural variation rather than definitive proof of complete crystalline–amorphous transformation.

Under the condition of a fixed OVA–TA complex concentration of 1.5% *w*/*v*, the surface morphology of the samples underwent significant changes as the XG concentration increased from 0.2% *w*/*v* to 1.0% *w*/*v*. At low XG concentrations (0.2% *w*/*v*–0.4% *w*/*v*), the surface exhibited extensive peeling, pores, and debris, with a rough and fragile structure, indicating that the polymer network had not yet formed effectively. When the XG concentration reached approximately 0.6% *w*/*v*, pore structures diminished, interlayer connectivity improved, and the surface became more continuous, suggesting that XG began to form a relatively tight encapsulating network [42]. At XG concentrations of 0.8% *w*/*v*–1.0% *w*/*v*, the surface appeared smoother with reduced debris, though minor edge peeling occurred occasionally, likely due to drying stress at higher concentrations [43]. On the other hand, under fixed XG concentration conditions, as the OVA–TA concentration increased from 0.5% *w*/*v* to 2.5% *w*/*v*, structural homogeneity first improved and then declined. At 0.5% *w*/*v* OVA–TA, the structure was loose with noticeable cracks; at 1.0% *w*/*v*–1.5% *w*/*v*, large-area peeling, pores, and debris were observed; at 2.0% *w*/*v*, structural fragmentation occurred, while at 2.5% *w*/*v*, the surface was smooth and uniform with minimal defects, representing the optimal morphology [44,45].

#### 3.1.3. Wettability Determination

**Figure 3:**
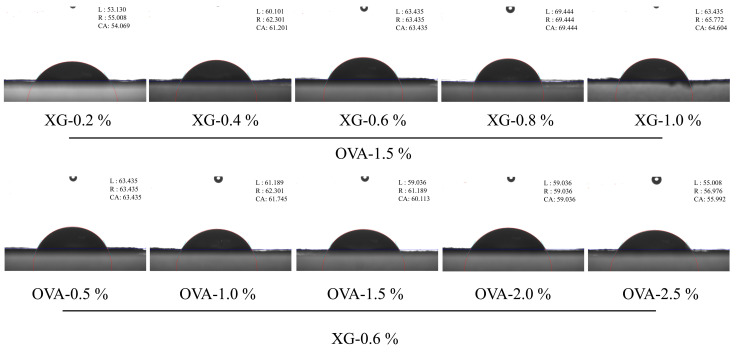
Surface wettability of OVA-TA-XG composite particles at different concentrations (characterized by contact angle). CA is the average value of the angle between the tangent of the left and right gas–liquid interfaces and the solid–liquid interface.

The three-phase contact angle of OVA–TA–XG composite particles at different concentrations reflects their surface wettability (Figure 3). Previous studies [46] indicate that particles with θ > 90° are relatively lipophilic, whereas those with θ < 90° are relatively hydrophilic. Particles with a contact angle close to 90° are generally considered favorable for strong adsorption at the oil–water interface and improved interfacial stability [47]. According to previous research, the contact angle of XG is 142.1°, whereas those of OVA–TA and OVA are both below 90°, indicating that XG exhibits stronger oleophilicity, while OVA–TA and OVA display greater hydrophilicity [48,49]. In the present study, the measured contact angles of the OVA–TA–XG complexes were below 90°, suggesting relatively hydrophilic characteristics. Therefore, the stabilization of the HIPEs should be interpreted as arising from the combined effects of interfacial adsorption, non-covalent interactions, continuous-phase network formation, and droplet packing rather than from classical Pickering stabilization alone.

When the OVA–TA concentration was maintained at 1.5% *w*/*v*, increasing the XG concentration from 0.2% to 1.0% *w*/*v* resulted in a non-linear change in the contact angle of the composite system, which first increased and then decreased, reaching a maximum of 69.444° at 0.8% *w*/*v* XG. Combined with FTIR and XRD analyses of OVA–TA–XG composite particles, this behavior may be related to changes in the balance between the relatively oleophilic XG component and the more hydrophilic OVA–TA component, as well as possible structural rearrangement or aggregation within the complexes. Conversely, when the XG concentration was fixed at 0.6% *w*/*v*, increasing the OVA–TA concentration from 0.5% to 2.5% *w*/*v* produced a complex trend in contact angle, characterized by an initial decrease, a subsequent increase, and a final decrease, reaching a minimum of 55.992° at 2.5% *w*/*v* OVA–TA. This phenomenon is consistent with the hydrophilic nature of OVA–TA, suggesting that higher OVA–TA concentrations may increase the overall hydrophilicity of the composite particles and lower their contact angle.

Overall, the OVA–TA-to-XG ratio plays an important role in regulating the surface wettability of the composite particles, which may further influence their interfacial behavior and the stability of the resulting HIPEs. However, because the contact angles were below 90° and direct interfacial measurements were not conducted, these results should be interpreted as indirect evidence of wettability regulation rather than definitive proof of classical Pickering stabilization. These findings provide useful guidance for designing OVA–TA–XG complex-stabilized HIPEs with improved emulsion stability.

### 3.2. Evaluation of OVA–TA–XG-Stabilized HIPEs

#### 3.2.1. Particle Size Analysis and Emulsion Type Analysis

**Figure 4:**
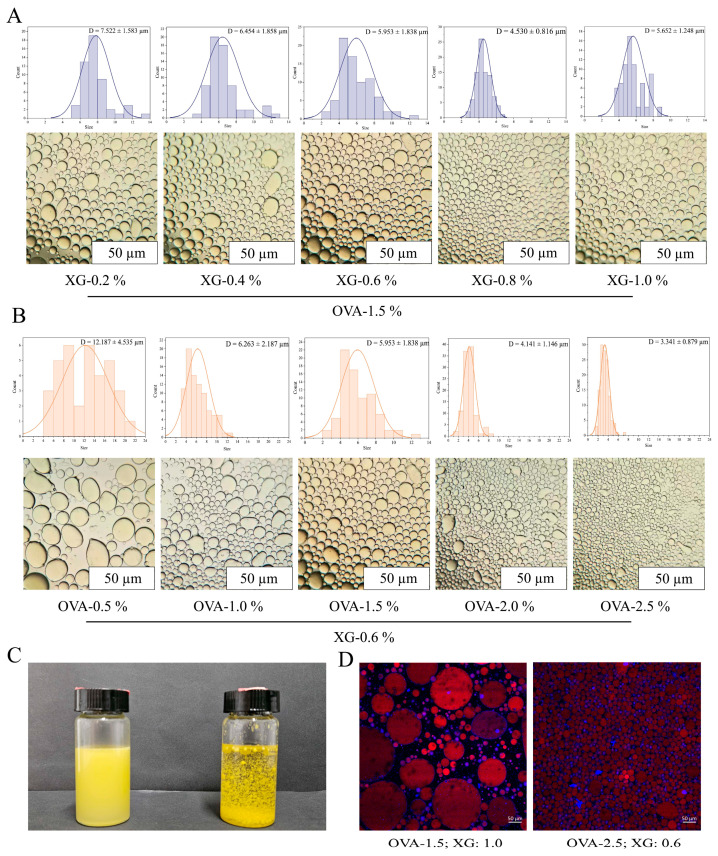
Particle size analysis and type determination of composite high-internal-phase emulsions with different concentrations. (**A**) Particle size analysis at fixed OVA–TA concentration with varying XG concentration. (**B**) Particle size analysis at fixed XG concentration with varying OVA–TA concentration. (**C**) Macroscopic appearance analysis. (**D**) CLSM analysis.

The droplet size first decreased and then increased with increasing XG concentration, reaching the minimum at 0.8% *w*/*v* XG. This suggests that moderate XG addition improved dispersion and interfacial/network stabilization, whereas excessive XG may promote chain entanglement or aggregation, resulting in larger droplets [50]. When XG was fixed at 0.6% *w*/*v*, increasing OVA–TA concentration continuously reduced droplet size, indicating enhanced interfacial coverage and suppression of droplet coalescence. CLSM and dispersibility analyses further confirmed that the OVA–TA–XG HIPEs were oil-in-water emulsions (Figure 4C,D), consistent with previous studies [48,51].

#### 3.2.2. Centrifugal Oil Retention and Auxiliary Emulsifying Properties

**Figure 5:**
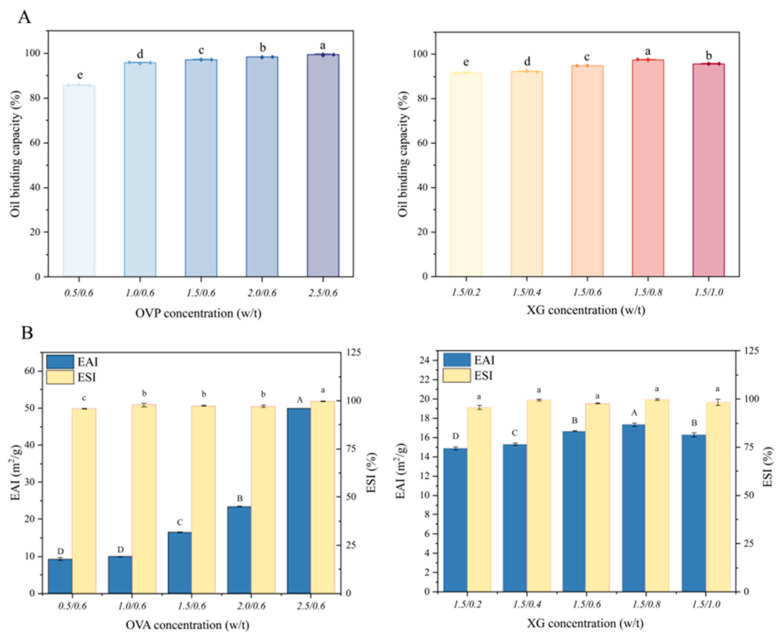
Centrifugal oil retention, emulsifying activity and stability of HIPEs. (**A**) Centrifugal oil retention. (**B**) Emulsifying activity and stability. Data represent means ± SD (*n* = 3). Different lowercase letters (a–e) denote significant differences (*p* < 0.05) among conventional ESI, whereas different uppercase letters (A–D) denote significant differences (*p* < 0.05) among EAI.

The ability of the HIPEs to retain oil under centrifugal stress was evaluated as a practical indicator of physical integrity and resistance to oil leakage. A higher oil-retention value indicates that the oil phase is more effectively immobilized within the droplet network and continuous phase structure, thereby reducing oil release or migration [52]. It should be noted that this parameter is used here as a practical oil-retention index rather than as a definitive descriptor of interfacial stability. As shown in Figure 5A, when the OVA–TA concentration was fixed at 1.5% *w*/*v*, the oil retention of the HIPEs increased with XG concentration up to 0.8% *w*/*v*, reaching 97.54 ± 0.18%, but decreased to 95.66 ± 0.08% at 1.0% *w*/*v* XG. This trend is consistent with the droplet size results, suggesting that moderate XG addition may have improved network formation and oil immobilization, whereas excessive XG may have caused aggregation or structural heterogeneity.

The formation of a compact droplet network and sufficient interfacial coverage is important for maintaining the physical stability of HIPEs, particularly under external stress. Poor interfacial/network stabilization may lead to droplet coalescence, phase separation, oil leakage, or undesirable texture changes [31]. When XG was fixed at 0.6% *w*/*v*, increasing OVA–TA concentration enhanced oil retention, which may be attributed to improved interfacial coverage and a more compact droplet network. However, the interpretation of oil retention should be considered together with other structural and rheological indicators, because HIPEs are highly concentrated systems whose stability depends on droplet packing, network strength, and bulk viscoelasticity [53].

EAI and ESI were measured as auxiliary indicators of the emulsifying capacity of the OVA–TA–XG systems after dilution. Because HIPEs are highly concentrated, non-dilute emulsions with closely packed droplets, these turbidity-based indices should not be interpreted as definitive measures of HIPE stability. Previous studies have shown that emulsifying activity and stability are often related to protein concentration in diluted emulsion systems [54,55]. As shown in Figure 5B, at a fixed XG concentration of 0.6% *w*/*v*, EAI increased with increasing OVA–TA concentration and reached 49.91 ± 0.06 m^2^/g at 2.5% *w*/*v*, indicating enhanced emulsifying capacity with higher protein–polyphenol complex concentration. In contrast, at a fixed OVA–TA concentration of 1.5% *w*/*v*, EAI showed no clear dose-dependent response to XG concentration and slightly decreased at 1.0% *w*/*v* XG, possibly due to excessive polysaccharide-induced aggregation. ESI remained relatively high among the tested formulations, but its interpretation should be considered together with droplet size, CLSM, rheology, and visual stability results.

Overall, these results indicate that appropriate OVA–TA and XG concentrations contributed to improved oil retention and auxiliary emulsifying performance. However, the stability of the HIPEs should be evaluated based on multiple structural and rheological indicators, including droplet size, microstructure, viscoelasticity, and storage/thermal stability, rather than relying solely on oil-retention capacity, EAI, or ESI [31,56].

#### 3.2.3. Rheological Properties

The rheological properties of HIPEs are closely related to their processing performance and potential application as fat replacers in biscuits. HIPEs with sufficient viscosity and elasticity can provide structural support, improve dough handling, and contribute to texture formation, whereas excessively weak or unstable systems may impair product quality [57]. Therefore, the viscoelastic properties and flow behavior of OVA–TA–XG-stabilized HIPEs with different XG or OVA–TA concentrations were investigated.

**Figure 6:**
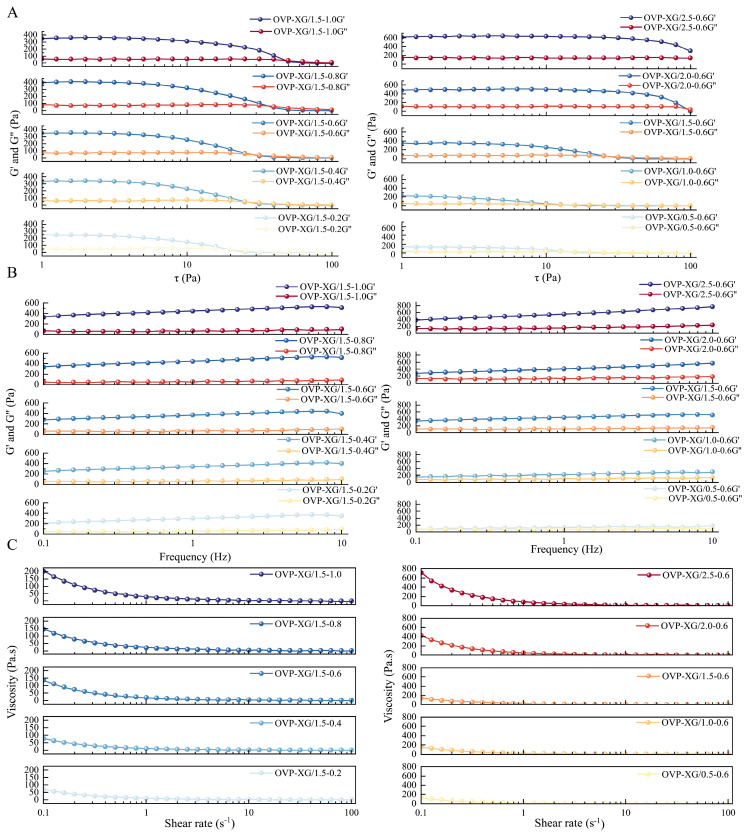
Rheological properties of HIPES. (**A**) Amplitude sweep. (**B**) Frequency sweep. (**C**) Viscosity profile. Data represent means ± SD (*n* = 3).

The formation of HIPE structures was supported not only by the internal oil phase fraction of 75%, but also by structural and rheological evidence. Optical microscopy and CLSM observations showed closely packed oil droplets within the continuous aqueous phase (Figure 4C,D). In addition, the presence of a linear viscoelastic region followed by structural yielding (Figure 6A), together with elastic-dominated behavior where G′ remained higher than G″ (Figure 6B), further supports the formation of a jammed and viscoelastic droplet network.

As shown in Figure 6A, all samples exhibited a linear viscoelastic region (LVR) at low stress, where G′ and G″ remained relatively stable, indicating that the internal network structure was maintained. With increasing stress, both moduli decreased sharply, suggesting structural yielding. A wider LVR and higher resistance to deformation generally indicate stronger structural integrity during processing [58]. Increasing XG concentration broadened the LVR, suggesting that XG contributed to the formation of a stronger continuous-phase network and improved shape retention. However, the yield-related response did not increase continuously with XG concentration; instead, the best resistance to deformation was observed at approximately 0.8% *w*/*v* XG. This result is consistent with the droplet size and oil-retention results, suggesting that moderate XG addition favored droplet packing and network formation, whereas excessive XG may have caused structural heterogeneity or local aggregation.

Frequency sweep results further confirmed the gel-like behavior of the HIPEs (Figure 6B). For all formulations, G′ remained higher than G″ over the entire frequency range, indicating that the elastic component dominated and that the emulsions possessed solid-like characteristics [59]. Increasing either XG or OVA–TA concentration generally increased G′, suggesting enhanced network strength and gel elasticity. This may be attributed to the combined effects of improved interfacial coverage by OVA–TA complexes and thickening/network formation by XG, which is consistent with previous studies on protein–polysaccharide-stabilized emulsion systems [48,60].

The steady shear results showed that all HIPEs exhibited typical shear-thinning behavior, with apparent viscosity decreasing as the shear rate increased (Figure 6C). This behavior is beneficial for processing because the emulsions can maintain structure at rest while becoming easier to deform or spread under shear. The apparent viscosity increased markedly with increasing XG concentration, mainly due to the thickening effect of XG in the aqueous phase and its contribution to network formation [60,61]. At a fixed XG concentration of 0.6% *w*/*v*, increasing OVA–TA concentration produced a moderate increase in initial apparent viscosity, which may be related to enhanced interfacial coverage, smaller droplet size, and stronger droplet–droplet interactions. However, excessive polysaccharide or biopolymer aggregation may also induce structural heterogeneity, which should be considered when optimizing the formulation [49]. Similar relationships between interfacial biopolymer layers, emulsion microstructure, and rheological behavior have been reported previously [62].

Overall, the rheological results indicate that OVA–TA–XG-stabilized HIPEs exhibited elastic-dominated and shear-thinning properties, supporting their potential use as structured fat replacers in biscuit formulations. Among the tested systems, moderate XG addition and sufficient OVA–TA concentration favored the formation of a stronger viscoelastic network. Nevertheless, the rheological behavior should be interpreted together with droplet size, microstructure, oil-retention capacity, and storage stability rather than as an isolated indicator of HIPE performance [15].

#### 3.2.4. Storage, Heating, and Freeze–Thaw Stability

**Figure 7:**
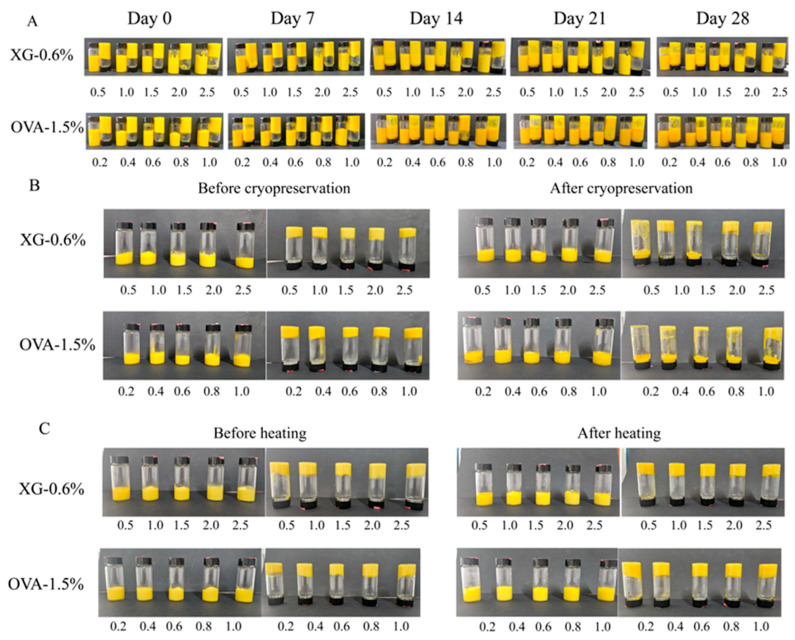
Visual observation of HIPE stability at various concentrations. (**A**) Storage stability. (**B**) Freeze–thaw stability. (**C**) Thermal stability.

Figure 7A illustrates the storage behavior of OVA–TA–XG-stabilized HIPEs over 28 days. During the first 7 days, emulsions containing 0.5–0.6% *w*/*v* and 1.5–0.2% *w*/*v* exhibited flow upon inversion. As the storage period extended to 14–28 days, most systems showed increased flowability, with the exception of the 2.0–0.6% *w*/*v* and 2.5–0.6% *w*/*v* formulations, which maintained only slight flow. Comparisons with the 1.5% *w*/*v* OVA-based system revealed that the 1.5–0.8% *w*/*v* formulation demonstrated the highest storage stability as XG concentration increased from 0.2% to 0.8% *w*/*v*, suggesting that elevating the composite concentration within an appropriate range strengthens the emulsion structure. However, further increasing XG content to 1.0% *w*/*v* did not yield additional improvement, likely because excessive polysaccharide levels induce elevated osmotic pressure, thereby promoting particle flocculation. This interpretation aligns with findings by Zhu et al., who reported that high polysaccharide concentrations generate osmotic pressure gradients between adjacent particles, driving aggregation [63]. Although the resulting viscosity increase may partially restrict flow, it cannot fully compensate for structural destabilization, leading to poorer inversion resistance than formulations with lower XG content. As shown in Figure 7B, all samples experienced oil–water separation after three freeze–thaw cycles, with pronounced phase separation observed in most systems except for 2.0–0.6% *w*/*v* and 2.5–0.6% *w*/*v*, which displayed only minimal droplet flow. In contrast, the thermal stability results in Figure 7C show no obvious phase separation or flow in any formulation during post-cycle inversion, indicating strong structural recovery under alternating stress.

Taken together, these observations support the conclusion that within a certain concentration window, increasing the concentration of the OVA–TA–XG composite reinforces the interior network and interfacial stability of the HIPEs, thereby improving its stability under static storage and inverted-gravity conditions. This is consistent with previous reports showing that moderate increases in particle or biopolymer concentration can enhance viscosity and internal network formation in HIPE systems, thereby suppressing phase separation and flow [59,64]. However, although the HIPEs showed good storage and thermal stability, phase separation after repeated freeze–thaw cycles indicates limited suitability for frozen foods or products subjected to freeze–thaw processing. Further improvement of freeze–thaw stability, such as strengthening the interfacial layer, optimizing the continuous-phase network, or incorporating suitable cryoprotective components, will be necessary for frozen product applications.

#### 3.2.5. Oxidative Stability

**Figure 8:**
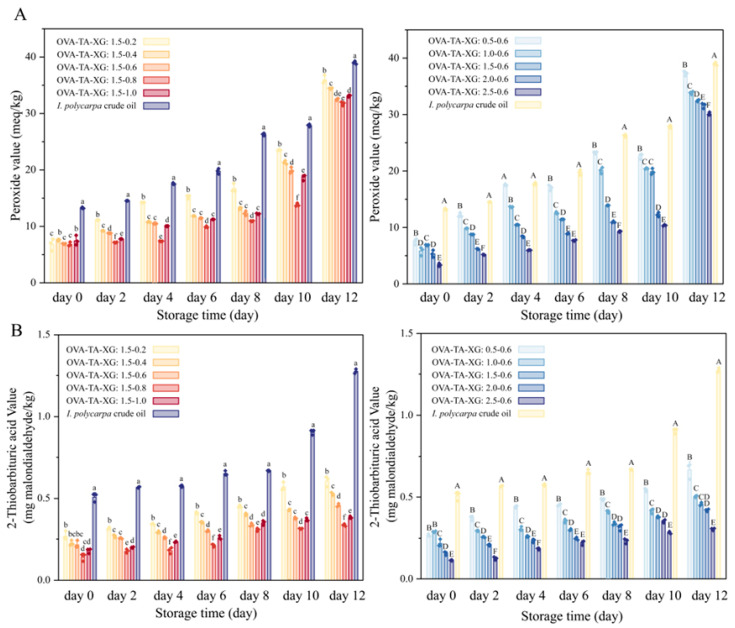
Evaluation of HIPE oxidative stability. (**A**) Initial peroxide value determination. (**B**) Secondary peroxide 2-Thiobarbituric acid value. Data represent means ± SD (*n* = 3). Different lowercase letters (a–f) indicate significant differences among HIPEs prepared with varying XG ratios while keeping the OVA–TA ratio constant (*p* < 0.05), and different uppercase letters (A–F) indicate significant differences among HIPEs prepared with varying OVA–TA ratios while keeping the XG ratio constant (*p* < 0.05).

The oxidative stability of the HIPEs was evaluated by measuring peroxide values and secondary oxidation products during accelerated oxidation at 60 °C for 12 days. As shown in Figure 8A,B, both peroxide values and secondary oxidation products of all samples increased with prolonged oxidation time. Among all samples, IPCO consistently exhibited the highest peroxide values and secondary oxidation products throughout the entire time course, indicating its high susceptibility to oxidation in the absence of stabilizers. When IPCO was encapsulated by OVA–TA–XG composite particles, the oxidation rate was significantly reduced. At a fixed XG concentration, increasing the OVA–TA concentration (with TA:OVA fixed at 1:10) improved the oxidative stability of the HIPEs. However, it should be noted that within this experimental design, TA was varied concomitantly with OVA at a fixed ratio; therefore, while the overall effect of the OVA–TA complex can be assessed, the individual contributions of OVA and TA to oxidation inhibition cannot be distinguished. Previous studies have suggested that TA, as a natural polyphenol, may possess antioxidant activity through radical scavenging and metal ion chelation [65,66], but confirming its specific role in this system would require comparison with TA-free controls. Consistent with CLSM observations, OVA–TA–XG particles formed a dense interfacial layer around oil droplets, which may help limit oxygen diffusion into the oil phase, thereby retarding lipid oxidation. At a fixed OVA–TA concentration, increasing the XG concentration resulted in a “first increase then decrease” trend in antioxidant performance, with the optimal effect observed at 0.8% *w*/*v* XG. Although 1.0% *w*/*v* XG did not further improve the antioxidant effect, its performance remained superior to that of lower concentrations (0.2%, 0.4%, and 0.6% *w*/*v*). This phenomenon may be attributed to the disruptive effect of excess XG on the composite particle structure, as excessive polysaccharide levels can increase osmotic pressure within the system, promoting particle aggregation and thereby compromising HIPE stability. This observation aligns with the findings of Wang et al., who reported that excess κ-carrageenan could disrupt the continuous protein network and induce aggregation of microgel particles and oil droplets [67].

Nevertheless, the high viscosity of XG may limit oxygen permeation, thus maintaining relatively strong antioxidant performance even at higher concentrations. However, it must be emphasized that the current experimental design lacks controls including HIPEs stabilized by OVA alone, OVA–XG without TA, and OVA–TA without XG, making it impossible to definitively distinguish between the chemical antioxidant effect of TA, the physical barrier effect of the interfacial layer, and the viscosity-mediated effect of XG. Future studies incorporating these control groups are necessary to fully elucidate the contribution mechanisms of each component to oxidative stability. Overall, these results underscore the importance of balancing OVA–TA and XG concentrations to optimize the oxidative stability of HIPEs, while acknowledging the need for additional controls to address component-specific contributions.

#### 3.2.6. Stabilization Mechanism of OVA-TA-XG HIPEs

**Figure 9:**
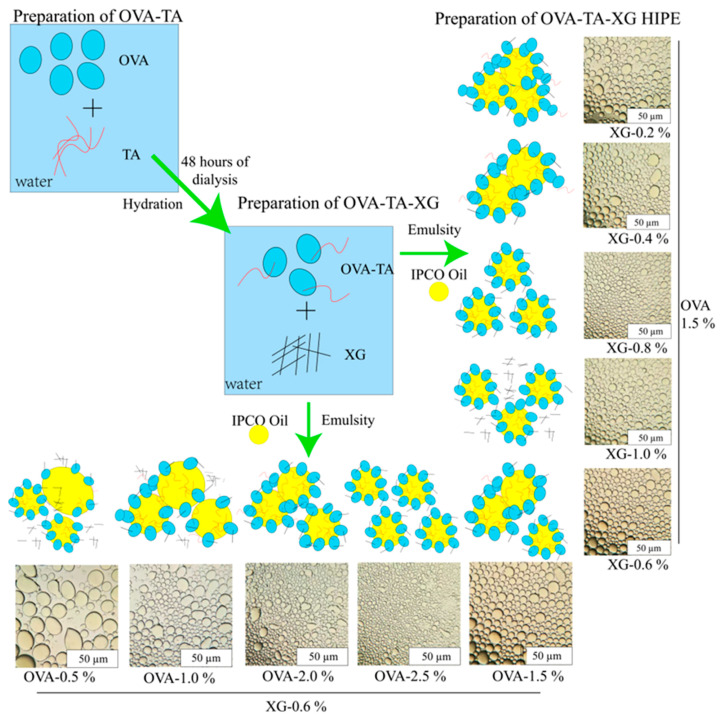
Mechanism of OVA-TA-XG composite HIPEs: OVA-TA-XG is synthesized by either fixing the OVA-TA concentration and adjusting the XG concentration (0.2–1.0% *w*/*v*) or fixing the XG concentration and adjusting the OVA-TA concentration (0.5–2.5% *w*/*v*). OVA: ovalbumin; TA: tannic acid; XG: xanthan gum; IPCO: *Idesia polycarpa* crude oil.

Based on the above results, a proposed stabilization mechanism of OVA–TA–XG-stabilized HIPEs is illustrated in Figure 9. It should be noted that although the OVA–TA–XG complexes contributed to HIPE stabilization, the present study did not directly demonstrate irreversible adsorption of rigid solid particles at the oil–water interface. Moreover, the measured contact angles of the complexes were below 90°, indicating relatively hydrophilic characteristics. Therefore, the HIPEs in this study are described as OVA–TA–XG-stabilized HIPEs rather than classical Pickering HIPEs. Their stabilization is proposed to arise from the combined effects of interfacial adsorption of OVA–TA complexes, non-covalent interactions involving XG, continuous-phase thickening/network formation, and droplet packing. At pH 7.0, both OVA and XG are expected to carry net negative charges; therefore, electrostatic attraction is unlikely to be the dominant driving force. Instead, hydrogen bonding, hydrophobic interactions, chain entanglement, and possible local patch–charge interactions may contribute to the association between OVA–TA complexes and XG.

Moderate XG addition improved droplet dispersion, network strength, and emulsion stability, as reflected by the reduced droplet size, enhanced oil retention, and stronger viscoelastic response. When XG concentration increased within an appropriate range, XG could participate in the formation of a continuous-phase network and restrict droplet movement. However, excessive XG may promote self-association, chain entanglement, or structural heterogeneity, leading to increased droplet size and reduced stability. This interpretation is consistent with the droplet size results, where the average droplet size decreased to 4.530 ± 0.816 μm at 0.8% *w*/*v* XG but increased to 5.652 ± 1.248 μm at 1.0% *w*/*v* XG. Similar concentration-dependent effects of XG on protein-based systems have been reported previously [50]. Excessive non-adsorbed polysaccharides may also induce depletion flocculation or local aggregation, which has been observed in OVA–XG-stabilized emulsion systems [68].

In contrast, increasing OVA–TA concentration may enhance interfacial coverage and contribute to the formation of a more compact droplet network. OVA, as an amphiphilic protein, can adsorb at the oil–water interface, whereas TA may participate in non-covalent interactions with OVA and provide antioxidant effects at or near the interface. XG may further reinforce the system by increasing continuous-phase viscosity and strengthening the surrounding network through hydrogen bonding and chain entanglement. Similar contributions of hydrocolloids to interfacial and rheological stabilization in protein-based emulsions have been reported previously [69].

It should be emphasized that the present study did not include direct interfacial measurements, such as zeta potential, interfacial tension, or interfacial rheology, nor did it include OVA-only, OVA–XG, and OVA–TA control systems. Therefore, the mechanism proposed here should be regarded as an interpretation based on indirect evidence from droplet size, CLSM, oil retention, rheology, oxidative stability, and visual stability results, rather than as definitive proof of a single stabilization pathway. Future studies incorporating direct interfacial characterization and additional control systems are needed to further clarify the specific contributions of OVA, TA, and XG to HIPE stabilization.

### 3.3. Evaluation of OVA-XG-TA HIPES Cookies

#### 3.3.1. Colorimetric Analysis of Biscuits

**Figure 10:**
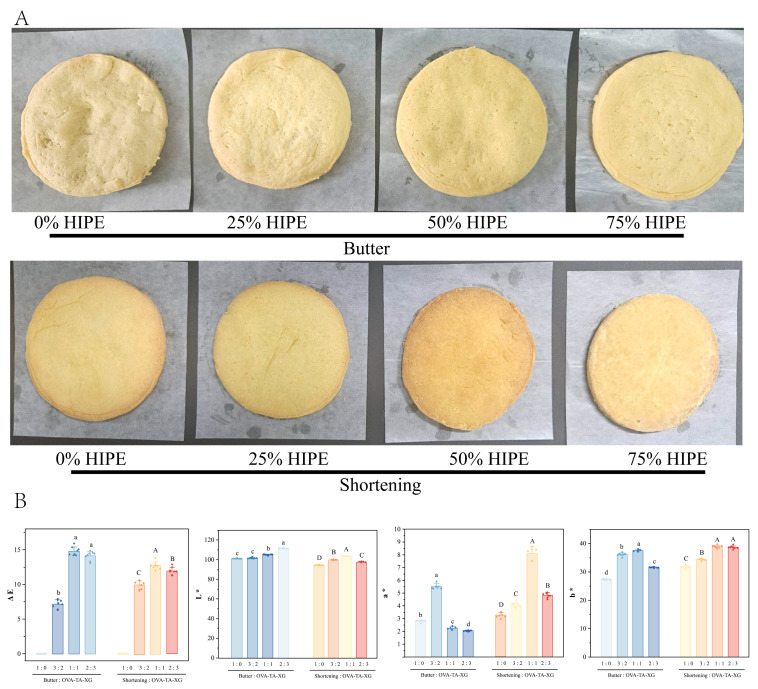
Evaluation of biscuit quality. (**A**) Visual characteristics. (**B**) Color parameters. Data represent means ± SD (*n* = 6). Different lowercase letters (a–d) indicate significant differences between traditional butter, butter, and HIPE-blended biscuits (*p* < 0.05), while different uppercase letters (A–D) indicate significant differences between traditional shortening, shortening, and HIPE-blended biscuits (*p* < 0.05).

As shown in Figure 10, when HIPEs stabilized by OVA-TA-XG ternary complexes were incorporated into the cookie system, the samples exhibited a consistent chromatic trend as the HIPE content increased: the lightness (L*) slightly increased or remained at relatively high levels, suggesting that the appearance became brighter; meanwhile, both a* and b* values shifted positively, indicating enhanced red and yellow tones that imparted a warmer hue to the cookies. Previous studies have noted that the total color difference (ΔE) is often used to evaluate whether visual differences between samples are perceptible to the human eye [70]. In this study, ΔE increased significantly with higher HIPE levels, particularly in the high-substitution group (e.g., 75% *w*/*w* HIPEs), where ΔE was markedly higher than in the low-substitution or control groups, demonstrating that higher HIPE incorporation leads to noticeable visual color differences. Therefore, although the addition of HIPEs may provide benefits in terms of texture and flavor, their pronounced influence on product appearance should be carefully considered in practical applications.

#### 3.3.2. Texture Analysis and Baking Loss of Biscuits

**Figure 11:**
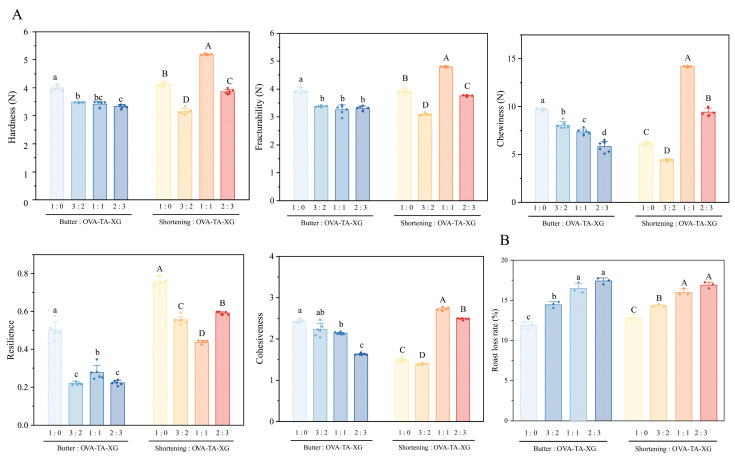
Biscuit texture and loss rate determination. (**A**) Biscuit texture analysis. (**B**) loss rate texture analysis. Data represent means ± SD (*n* = 3). Different lowercase letters (a–d) indicate significant differences between traditional butter, butter, and HIPE-blended biscuits (*p* < 0.05), while different uppercase letters (A–D) indicate significant differences between traditional shortening, shortening, and HIPE-blended biscuits (*p* < 0.05).

The results, as shown in Figure 11, demonstrated that replacing butter or shortening with protein–polysaccharide–polyphenol composite structured emulsions had significant effects on the textural properties of cookies, and exhibited typical nonlinear trends. When butter was used as the control, increasing replacement levels (25% *w*/*w*, 50% *w*/*w*, 75% *w*/*w*) led to a general decline in hardness, brittleness, chewiness, cohesiveness, and resilience. This phenomenon can be attributed to the abundant β′-type fat crystals in butter, which form a fine crystalline network and confer plasticity. In contrast, the structured emulsion relies mainly on protein–polysaccharide–polyphenol interactions (hydrogen bonding, hydrophobic interactions, and polyphenol cross-linking) to form its network, which is less dense and mechanically weaker, thereby failing to fully substitute the crystalline fat matrix and resulting in reduced textural performance [32,71].

In contrast, when shortening was replaced, a “decrease–increase–decrease” pattern was observed. At low replacement levels (25% *w*/*w*), the crystalline fat network of shortening was partially disrupted, while the newly formed composite emulsion network was insufficient to provide structural support, leading to significant reductions in hardness, brittleness, chewiness, and cohesiveness [72,73]. At intermediate replacement levels (≈50% *w*/*w*), a synergistic effect was observed between the fat crystal network and the protein–polysaccharide–polyphenol composite network. Polyphenols reinforced interfacial films through hydrogen bonding and hydrophobic interactions, resulting in more uniform oil droplet distribution and a denser, more balanced porous structure, thereby producing optimal textural properties [74,75]. However, at high replacement levels (75% *w*/*w*), the fat crystal network was almost completely lost, and the emulsion-based network tended to collapse during baking due to water evaporation, leading again to a decline in hardness, brittleness, chewiness, and cohesiveness [76].

It is noteworthy that resilience consistently decreased with increasing replacement level, regardless of whether butter or shortening was replaced. This is because fat crystal networks are partially reversible upon compression, while emulsion-templated colloidal networks are more prone to irreversible rupture under stress, lacking elastic recovery capacity. Consequently, the overall resilience of the system progressively weakened, which is consistent with previous reports on the elasticity of structured lipids [77,78]. In summary, butter replacement showed a monotonic decline in textural properties, whereas shortening replacement revealed a synergistic window at approximately 50% *w*/*w*, reflecting the distinct crystallization characteristics and structural contributions of the two conventional solid fats.

When replacing shortening and butter with emulsions, a 25% *w*/*w* substitution level can provide sufficient structural support, preserve the dough’s layering and air permeability, and thereby reduce baking loss. However, at higher substitution levels of 50% and 75% *w*/*w*, excessive emulsion replacement compromises the dough’s structural stability, disrupts the continuity of the fat layer, and increases baking loss, primarily due to the evaporation of water from the emulsion during baking [58].

#### 3.3.3. Fatty Acid Composition of Biscuits

Because no internal standard was used in the GC–MS analysis, the fatty acid data are presented as relative composition based on peak area normalization and should be interpreted as semi-quantitative results. In this study, increasing the replacement ratio from 0% to 75% *w*/*w* led to a systematic shift in the relative fatty acid composition in both the butter and shortening replacement series. Specifically, total saturated fatty acids (SFAs) decreased substantially—for instance, C16:0 (palmitic acid) declined from ~47.26% to 23.79% in the butter series and from ~57.47% to 30.71% in the shortening series—while total polyunsaturated fatty acids (PUFAs) increased markedly, with C18:2n6c (linoleic acid) rising from ~13.33% to 42.70% and from ~7.43% to 38.63% in the butter and shortening series, respectively. Monounsaturated fatty acids (MUFAs) also exhibited a gradual decline, although less pronounced. These relative compositional changes indicate that partial replacement with IPCO-based HIPEs shifted the lipid source contribution of the final biscuits from a saturated-fat-dominant profile toward a higher proportion of unsaturated fatty acids. Such findings are consistent with the growing body of literature emphasizing that partial replacement of saturated fats with more unsaturated lipid systems—including vegetable oils, oleogels, polymeric or hydrogel matrices—represents a viable strategy for improving lipid healthiness and reducing cardiovascular risk. These findings are consistent with previous reports showing that structured unsaturated lipid systems can be used as fat replacers in baked products to improve lipid composition while maintaining acceptable product quality at appropriate substitution levels [79]. Taken together, our results highlight that a replacement level within the 50–75% *w*/*w* window offers a promising balance: significant improvements in the unsaturated fatty acid content while maintaining acceptable texture and sensory quality. Future research should further assess oxidative stability, flavor profile, and storage performance to provide a more comprehensive evaluation of this fat replacement strategy in cookies and related bakery applications. 

Compared with oleogels, which generally mimic solid fats through crystalline or polymeric oil networks, HIPEs provide an alternative structuring strategy by immobilizing oil droplets within a concentrated emulsion network. In this study, OVA–TA–XG-stabilized HIPEs enabled partial replacement of butter or shortening while maintaining relatively comparable biscuit texture at selected substitution levels and increasing the relative proportion of unsaturated fatty acids in the final products. Nevertheless, compared with more extensively studied oleogel and commercial fat-replacement systems, the current HIPEs still require further validation in terms of sensory quality, shelf-life performance, processing tolerance, and overall practical competitiveness.

### 3.4. Limitations of This Study

This study has several limitations. The TA:OVA mass ratio was fixed at 1:10, and TA was not independently varied; moreover, OVA-only, OVA–XG, and OVA–TA control systems were not included. Therefore, the individual contributions of OVA, TA, and XG cannot be fully distinguished. Although a threshold effect of XG was observed, whether OVA–TA exhibits a similar threshold remains unclear. In addition, direct interfacial measurements, such as zeta potential, interfacial tension, and interfacial rheology, were not performed; thus, the proposed stabilization mechanism was inferred from indirect evidence, including structural, rheological, oxidative stability, and visual stability results. Finally, the freeze–thaw stability of the HIPEs was limited, and the biscuit application lacked sensory evaluation and shelf-life tests. Future studies should include direct interfacial characterization, additional control groups, sensory evaluation, and storage tests to validate consumer acceptability, as well as comparisons with other fat replacers such as oleogels or commercial alternatives.

## 4. Conclusions

This study developed a strategy to stabilize IPCO-based HIPEs using OVA–TA–XG complexes, thereby delaying IPCO oxidation and enabling partial substitution of conventional solid fats in biscuits. Structural analyses (FTIR, XRD, SEM, CLSM) confirmed strong non-covalent interactions among the components and dense interfacial adsorption of the composite particles at the oil–water interface. It should be noted that in this experimental design, TA and OVA were varied together at a fixed ratio of 1:10 based on preliminary optimization; therefore, while the combined effect of the OVA–TA complex can be evaluated, the individual contributions of OVA and TA to the observed structural and functional properties cannot be dissociated. Among the formulations tested, the HIPEs prepared with 2.5% *w*/*v* OVA–TA (with TA:OVA fixed at 1:10) and 0.6% *w*/*v* XG exhibited optimal performance in terms of small particle size (3.314 μm), high centrifugal oil retention (99.29%), auxiliary emulsifying activity index after dilution (49.91 m^2^/g), and auxiliary emulsifying stability index after dilution (99.69%), and was therefore selected for subsequent biscuit application trials. In biscuits, replacing 25% *w*/*w* of butter or 50% *w*/*w* of shortening with this optimized HIPEs achieved texture properties close to the controls. GC–MS analysis showed that palmitic acid (C16:0) decreased from approximately 47.26% to 23.79% in the butter replacement series and from approximately 57.47% to 30.71% in the shortening replacement series, while linoleic acid (C18:2n6c) increased to 42.70% and 38.63%, respectively. Overall, OVA–TA–XG-stabilized HIPEs effectively delayed IPCO oxidation and, through partial replacement of conventional solid fats, shifted the relative fatty acid composition of the final bakery products toward a lower relative proportion of saturated fatty acids and a higher relative proportion of polyunsaturated fatty acids, providing new opportunities for the food application and industrial development of IPCO.

## Data Availability

The original contributions presented in the study are included in the article; further inquiries can be directed to the corresponding author.
